# From antiquity to modern hygiene: the archaeological and medicinal legacy of lavender as a promising antimicrobial agent

**DOI:** 10.3205/dgkh000550

**Published:** 2025-05-20

**Authors:** Agne Civilyte, Kyriaki Karanikola, Axel Kramer

**Affiliations:** 1 Department of Archaeology, Lithuanian Institute of History, Vilnius, Lithuania; 2 Department of Archaeology, University of Vilnius, Vilnius, Lithuania; 3 Institute of Hygiene and Environmental Medicine, University Medicine Greifswald, Greifswald, Germany

**Keywords:** lavender, essential oils, roots ancient world, antimicrobial efficacy, perspectives for development

## Abstract

**Introduction:**

The understanding of ancient medicinal and hygienic practices using medicinal plants provides a foundation for collaboration with modern medical science. The aim of this paper is first to give a review of archaeological evidence of one of such plants – lavender, belonging to the Lamiaceae family, particularly focusing on its application for hygienic purposes. Since lavender is notable for its pharmacological and medicinal properties, offering a wide range of benefits for both physical and mental health, this is worth combining ancient experiences with the research of modern medicine.

**Methodology:**

The historical applications of lavender from antiquity to the Middle Ages were analyzed both textual and archaeobotanical evidence. Furthermore, it discusses the challenges in detecting lavender in archaeological contexts and its potential contributions to modern antiseptic medicine and heritage conservation. By combining botanical studies with modern scientific methods, we aim to reassess its role in historical medical and hygienic practices.

**Results:**

In the last two decades, following on from traditional knowledge from archaeology and antiquity, studies have been carried out on the antimicrobial effectiveness of lavender essential oils (LEO) with the result that there are potential applications for LEO for antiseptic, as combination partner in disinfectants, for preservation, impregnation and possibly also for decontamination of indoor air. Even more promising is the use of LEO in combination with antibiotics and antiseptics in order to synergistically enhance their effectiveness.

**Conclusion:**

By combining botanical studies with modern scientific methods, the role of lavender in historical medical and hygienic practices has been re-evaluated.

## Zusammenfassung

**Hintergrund:**

Das Verständnis antiker medizinischer und hygienischer Praktiken unter Verwendung von Heilpflanzen bietet eine Grundlage für die Umsetzung relevanter Erkenntnisse in die moderne medizinische Wissenschaft. Im Artikel soll ein Überblick über die archäologischen Belege für Lavendel mit besonderem Augenmerk auf seine Verwendung für hygienische Zwecke gegeben werden. Da Lavendel für seine pharmakologischen und medizinischen Eigenschaften bekannt ist und eine breite Palette von Vorteilen für die körperliche und geistige Gesundheit bietet, lohnt es sich, antike Erfahrungen mit der Forschung der modernen Medizin zu verbinden.

**Methodologie:**

Es wurden die historischen Anwendungen von Lavendel von der Antike bis zum Mittelalter untersucht, wobei sowohl textliche als auch archäobotanische Belege analysiert werden. Darüber hinaus werden die Herausforderungen beim Nachweis von Lavendel in archäologischen Kontexten und sein potenzieller Beitrag zur modernen Medizin und zur Erhaltung des kulturellen Erbes erörtert.

**Ergebnisse:**

In den beiden letzten Jahrzehnten wurden anknüpfend an überliefertes Wissen aus der Archäologie und Antike Untersuchungen zur antimikrobiellen Wirksamkeit von ätherischen Ölen aus Lavendel (LEO) mit dem Ergebnis durchgeführt, dass sich für LEO Anwendungsmöglichkeiten zur Antiseptik, als Kombinationspartner in Desinfektionsmitteln, zur Konservierung, Imprägnierung und möglicherweise auch zur Dekontamination der Raumluft ergeben. Noch aussichtsreicher ist der Einsatz von LEO in Kombination mit Antibiotika und Antiseptika, um deren Wirksamkeit synergistisch zu verstärken.

**Schlussfolgerung:**

Durch die Kombination botanischer Studien mit modernen wissenschaftlichen Methoden wurde die Rolle des Lavendels in historischen medizinischen und hygienischen Praktiken neu bewertet.

## Introduction

Natural products extracted from plants play a crucial role in treating various diseases in modern medicine. In 1989, the World Health Assembly published the resolution 42.43 on traditional medicine and modern health care. As a result, the Word Health Organization developed the guidelines for the Assessment of Herbal Medicines in 1991 [1].

The use of plants and essential oils for medicinal and hygienic purposes dates back to ancient times. Writings from Ancient Egypt, along with Greek and Roman authors [2], [3], highlight the significance of botanical resources in healthcare and healing practices. Archaeologists discover paleobotanical remains of ancient plants and are able to analyze the substances used in ancient times.

Lavender, belonging to the Lamiaceae family, is one such plant with a long history of use for hygienic, medicinal, and aromatic purposes. As new treatment methods using natural products are being developed for human health, lavender’s low cost and minimal adverse effects make it particularly valuable. Various pharmacological properties have been identified, including antioxidant, antibacterial, anxiolytic, analgesic, and anti-inflammatory effects [4], [5]. 

Despite its numerous documented benefits, lavender remains an underexplored topic in archaeological and historical research. Only a few articles provide insights into how lavender was historically used for hygienic and medicinal purposes. Most of the available information is published on the websites of companies (often without references) that produce lavender oil and perfumes [6], [7], [8].

Therefore, the history of lavender use is not as clear as it might initially appear. However, we believe that even certain inaccuracies in the information do not diminish the importance of investigating the archaeological evidence of lavender in ancient cultures. Notably, in archaeological times, lavender was used in two forms: as an herb, bound into what were known as “nards”, and as an oil.

## Lavender in ancient cultures: archaeological and textual evidence

Dating back to pre-Roman epochs, one of the most prominent examples of evidence for the use of lavender is associated with Tutankhamun’s tomb (c. 1341 BCE–c. 1323 BCE *[Before Common Era*]). Some containers with plant material were part of the inventory of the tomb [9]. However, research into lavender in this context frequently turns up a phrase that has become nearly axiomatic but lacks specific references: *“It’s said that the Egyptians used lavender in perfumes, and when Tutankhamun’s tomb was opened, traces of lavender were found, and its scent could still be detected”.* The original author of this information is unclear, but it is highly unlikely to be accurate. For example, in her book Lise Manniche states “When the tomb of Tutankhamun was cleared, a modest bowl of incense was found in it. The report suggests that this was indeed frankincense” [10]. Furthermore, lavender is never mentioned in this monograph – instead, it details the various oils and resins that were detected in the tomb – used for a well-established standard embalming procedures such as washing and anointing the body with sacred oils, separate embalming of internal organs and their placement in canopic jars [11]. The reasons for such inaccuracies may lie in the scarce documentation and the rudimentary level of chemical analysis available at the time of the tomb’s discovery.

More recent chemical investigations of embalming workshops from the 24^th^ Dynasty in Saqqara (664–525 BCE) further support the absence of lavender in Egyptian mummification practices [12]. Inscriptions with Hieratic and Demotic texts were found in an embalming workshop located just a few meters south of the pyramid of King Unas. These inscriptions provided embalming instructions (e.g., “to put on his head” or “bandage or embalm with it”) and listed names of embalming substances (e.g., “sefet” or “dry antiu”), sometimes accompanied by the title of an administrator of the embalming workshop or necropolis. 

Chemical residues in vessels from this workshop using organic residue analysis (ORA) were analyzed. This analysis provided completely new insights into the chemistry of embalming. As a result, a mixture of elemi, Pistacia resin, oil or tar of juniper/cypress and cedar, animal fat, beeswax, likely castor oil, and a plant oil (probably olive) was identified. This example demonstrates that even if Egyptian mummification was built upon and fostered long-distance exchange and routes, including imports from the Mediterranean, lavender was not included in the recipes for embalming mixtures. Therefore, this suggests that the widely held belief of lavender’s role in ancient Egyptian burials might stem from later misinterpretations rather than verifiable evidence. 

Lavender is also barely present in the ancient botanico-medical literature. The earliest written references to lavender appear in the works of Theophrastus (371–287 BCE *[Before Common Era*]) and Dioscorides (40–90 AD *[anno Domini nostri Iesu Christi]*). In the founding treatise of botany by Theophrastus [13], it is described as *iphyon* in only two lists: one of the plants growing from seeds (Historia Plantarum-Inquiry into Plants, chapter 6.6.11) and the other of those flourishing in summer (chapter 6.8.3) [14].

A more detailed though still very brief description of lavender defined as stoichas is given by the Greek physician Pedanius Dioscorides (40–90 AD) in his work “De Materia Medica”: a sweeping 5-volume encyclopedic work of herbal medicine and related medicinal substances [2]. The work was written between 50 and 70 AD. It was widely read for more than 1,500 years until supplanted by revised herbals in the Renaissance, making it one of the longest-lasting of all natural history books. He recorded the ingredients and recipes used by Egyptian perfumers for posterity. Dioscorides gives a very scarce characterization of *stoichas:*

1. It grows in the islands below Gaul, in front of Massalia (Marseille), which are called *stoichas* – this is where it took its name from;
2. It is an herb with a thin seed, with a foliage similar to that of thyme, with, however, longer leaves, a pungent taste, somewhat bitter;
3. Its decoction is efficacious for chest conditions, like hyssop. It is usefully mixed in the antidotes.

Galen (129–99 AD) also mentions lavender as a remedy for poisonings and bites, suggesting the mixture of lavender with wine as an antidote for snake bites or stomach pains. Pliny the Elder (23–79 AD) cites it as a remedy for melancholy, attributing active properties for women and regulating menstruation, as well as for diluting expensive perfumes [3].

The Romans were instrumental in spreading lavender’s use across Europe, primarily for its aromatic and hygienic benefits [15]. Interestingly, the etymology of “lavender” is derived from the Latin “lavare,” meaning “to wash,” highlighting its role in Roman bathhouses. The antibacterial and antiviral properties of lavender-infused water contributed to sanitation and personal hygiene, further cementing its medicinal reputation.

In the Middle Ages, the use of lavender was also significant, as indicated by both written sources and archaeological findings. In the Iberian Peninsula, archaeobotanical remains of lavender have been found in Christian contexts as well as in Islamic contexts [16]. The flowering plants of the Lamiaceae family, such as lavender, were also used during these centuries in the care of the dead, as they appeared to have antiseptic properties.

Findings from Italy (15^th^–16^th^ century AD) indicate that lavender was used in the embalming process of mummies: *“All incisions must be filled with powder from various substances, such as: pomegranate powder, chamomile, sweet clover balm, mint, dill, sage, lavender, rosemary, oregano, and thyme”* [17]. Macro-remains of lavender have been also found in the cavities of the body of Saint Christina from Spoleto (ca. 1432–1458) [18].

The written sources on lavender and its therapeutic properties are numerous. For example, the abbess Hildegard (1098–1179) mentions that lavender is not meant to be consumed as food but that its strong scent can help with lice and evil spirits. The English botanist John Gerard (1545–1612 AD) describes various ways in which lavender can be used, whether for migraines or heart conditions [19].

## Challenges in detecting lavender in the archaeological record

Given lavender’s modern reputation for its antiseptic and soothing properties, we hypothesized that ancient Egyptians and Romans might have utilized lavender for hygiene-related practices. However, our research has revealed a lack of substantial evidence supporting lavender’s use in ancient Egypt and scarce documentation in Roman sources regarding its application in hygiene.

The absence of physical evidence for lavender in many archaeological sites does not necessarily indicate its absence from ancient societies. Several factors complicate its preservation and identification:

1. **Fragility of flowers and essential oils**: Unlike seeds or charred plant remains, flowers and oils degrade rapidly and rarely survive in archaeological contexts.
2. **Preservation conditions**: Macro-remains of lavender are unlikely to be preserved unless found in exceptionally arid, waterlogged, or anaerobic environments.
3. **Archaeobotanical analysis limitations**: Standard excavation techniques primarily focus on more resilient plant materials, leading to potential underrepresentation of lavender.

Archaeobotanical research focuses on the fragmentary remains of plants from archaeological sites, their taxonomic identification, and their interpretation concerning human activities and the environment [20]. A general division in archaeobotany distinguishes between macro-remains and micro-remains [21].

Macro-remains primarily include the study of seeds and wood charcoal. At the same time, micro-remains, such as phytoliths, pollen, and starch grains, are very small (<0.1mm) and therefore invisible to the naked eye It is important to note that these data represent a part of a larger collection of potential environmental datasets.

The preservation of the macro-remains archaeobotanical material at an archaeological site, and its subsequent discovery during excavations, can occur under specific conditions namely

1. Through charring, where plant parts come into contact with fire for a certain duration;
2. In waterlogged environments, where plant parts are preserved due to moisture;
3. In anaerobic conditions, where preservation occurs due to the absence of oxygen;
4. In some cases, plant remains can be maintained by mineralization.

In the first case, which is the most common in the Mediterranean, plant seeds are preserved in the soil due to the charring process. As such, the flowers of the plants are destroyed on-site (turning to ash), making it impossible for them to be preserved and found in the archaeobotanical record. In the second case, waterlogged plant species are preserved due to the moisture of the aquatic landscape; however, these conditions are not as frequent in the Mediterranean, where lavender primarily grew and was utilized. Therefore, with the third case of extreme aridity, where due to drought and lack of oxygen, plant species such as flowers can be preserved. Such conditions are typically found in Egyptian tombs.

Although lavender is a plant of the Old World that primarily grows in the drier climates of Eurasia, the discovery of its seeds in archaeobotanical assemblages from prehistoric times is not particularly common. Its use seems to have been initially associated with its aromatic and therapeutic properties, rather than as a food source. This indicates that people did not engage in intensive cultivation of this species, as it was not an immediate need, unlike other plant species that were used for food and cultivated in large quantities.

The *chaîne opératoire* of food production encompasses several stages: primary production, harvesting and initial processing, storage, post-storage processing, cooking, consumption, and disposal [20]. Each of these stages offers different opportunities for preservation in the archaeobotanical record and can be investigated using various types of evidence. However, the use of flowering plants, like lavender, is more closely related to the flowers (or leaves) of the plant and not the seeds. Specifically, the post-harvest process involved drying the flowers and subsequently extracting essential oil, which was then mixed with other materials depending on the desired product.

Considering all of the above, it is evident that lavender was not used in the same way as other plants, substantially reducing the chances of its preservation in archaeological deposits and its identification as macro-remains. However, the absence of lavender does not imply that it was not present. As mentioned, the anaerobic/arid conditions of Egyptian tombs helped preserve many plant species that were placed within them. 

Advances in scientific technology have provided additional methods for identifying plant traces in settlements as macro and micro-remains [20], [21]. Chemical analysis of vessels is one of these methods. Samples from the interiors of vessels are analyzed, providing insights into their last contents. 

Through chemical analysis of these types of vessels, which appear not to have been involved in food-related processes, a new path may open for discovering plant species that served different purposes in settlements and are nearly impossible to identify otherwise for the reasons already mentioned.

One such case is the prehistoric site (Bronze Age) of Pyrgos Mavroraki in Cyprus destroyed by an earthquake in 1900–1850 BCE. There, one of the excavation areas was identified by archaeologists as a workshop used for the production of aromatic and therapeutic essences [22]. Chemical analysis of the vessels revealed the species that were used last. In one of these (PY05/G9L4) the indication of colors was interpreted by specialists as lavender, confirming the hypothesis that this species was already in use during prehistoric times. 

## Future development opportunities on the foundations of antiquity

In summary, lavender has been utilized by humans for centuries. Its absence from archaeological contexts does not in any way imply that this plant went unnoticed by human activities. On the contrary, its many properties as a medicinal or aromatic plant seem to have made it particularly popular in the past. Further research on this topic could enrich our understanding of the use of these plants in societies, primarily through micro-remains (e.g., chemical analyses of ceramics). Consequently, lavender represents a plant of considerable historical and cultural importance, illuminating the complex interplay between humanity and the natural world throughout the centuries.

Despite the challenges in detecting its use, lavender remains an essential bridge between ancient botanical knowledge and modern scientific advancements. This reevaluation is crucial for accurately reconstructing historical botanical practices. Furthermore, it encourages a more delicate approach to integrating traditional knowledge with contemporary medical and hygienic practices, stressing the importance of historical context in the application of modern remedies. 

Due to the study situation at the time of publication of the German-language manual on sterilization, disinfection, antiseptics and preservation (984 pages), the antiseptic efficacy of lavender essential oil (LEO) was not considered [23]. Only in the last two decades, LEO has become the focus of antimicrobial drug research with the following findings. The antibacterial and antifungal efficacy has been proven in vitro [24], [25], [26], [27], [28], [29], [30], [31], [32], [33], [34]. Moon et al. [35] introduced their study with the following words: “Although there is considerable anecdotal information about the antibacterial activity of lavender oils, much of this has not been substantiated by scientific or clinical evidence”. The study supported the anecdotal use of lavender oils as antibacterial agents. LEO, hydrosols and aqueous and ethanolic foliage extracts from a range of Australian grown Lavandula demonstrated that some oils are antibacterial effective against *Streptococcus pyogenes*, *Staphylococcus (S.) aureus*, methicillin resistant *S. aureus* (MRSA), *Citrobacter freundii*, *Proteus (P.) vulgaris*, Es*cherichia coli*, vancomycin resistant enterococci (VRE) and *Cutibacterium acnes*; only *Pseudomonas aeruginosa* was not susceptible. The lavender hydrosols and aqueous foliage extracts did not have any antibacterial activity. Six of the ethanolic extracts displayed activity against *P. vulgaris* but no activity against any other organism. LEO is also effective against respiratory pathogens in biofilms of different maturity with highest efficacy if distilled before flowering [36]. Further work is required to determine whether these in vitro results will be realized in a clinical environment but it is clear that not all lavenders are equal in terms of their antibacterial properties. The study underlines how important it is to analyze knowledge from archaeology and antiquity with the possibilities of modern implementation.

Due to the broad spectrum of effects of LEO, the following applications are considered promising and recommended for in-depth investigation: Use as cake preserving agent [31], protection of food commodities/crops from harmful organisms [37], antimicrobial impregnation of paper [38] , spray-dried oregano oil and lavender oil microcapsules for antibacterial sports and leisurewear [39], incorporation in liquid soap [40] applying LEO or oil-emitted flavor in the patient waiting room to decontaminate the air along with mental relaxation [41]. To mitigate the risk of infection, a hemostatic chitosan based sponge with an antibacterial barrier against a wide range of gram-positive and Gram-negative microorganism was developed [42].

One of the current challenges in wound care is the development of multifunctional dressings that can both protect the wound from external agents and promote the regeneration of the new tissue. The combined use of two naturally derived compounds, sodium alginate and LEO, in engineered dressings reduce the risk of microbial infection of the burn, since they stop the growth of *S. aureus*. Furthermore, they are able to control and reduce the inflammatory response that is induced in human foreskin fibroblasts. The down-regulation of cytokines production and the absence of erythema on the skin of the treated animals confirm that the dressings are promising as advanced biomedical devices for burn management [43].

LEO-loaded nanogels had the potential to serve as effective option for the management of bacterial infections [44]. Nanosized LEO formulations with improved physicochemical properties and enhanced bioactivities can be employed in the food processing sector for preservation [45].

Due to the increase in multi-drug resistant organisms, more and more studies are being carried out to improve the effectiveness of antibiotics by combining them with LEO. For example, synergism with LEO has been demonstrated for ampicillin [46], Gentamycin [47], penicillin and tetracycline [48]. LEO induce oxidative stress in *K. pneumoniae* which oxidizes the outer membrane, enabling the influx of generated ROS, LeO and meropenem into the bacterial cells, causing damage to the cells and eventually death [49].

Studies on increasing the efficacy of antiseptics through such combinations are also becoming increasingly important. For LEO, it has been shown that it increases the efficacy of octenidine dihydrochloride (OCT) against MRSA strains, because OCT potentially influences bacterial permeation by modifying the cell wall structure [50]. 

One review concludes that LEO against *S. aureus* and MRSA in some studies is effective, while other studies reporting a minimal to negligible effect. But LEO acts synergistically with other antibacterial agents, thus additional research in this area would be beneficial [51]. 

Extensive research is being carried out worldwide to identify the chemical components of LEO, to identify the biologically active constituents and to determine any synergistic effects of the ‘mixed’ components and to clarify the mode of action [52]

In addition to the listed applications with regard to antimicrobial efficacy, LEO is also used in disinfectants and antiperspirants.

## Conclusions

There is significant potential to further analyze archaeological findings related to hygienic practices, such as bath and sanitation complexes, toiletry sets, and pottery used for storing oils. These artifacts can provide valuable insights into the daily hygiene routines of prehistoric societies, including the materials and substances they employed. Future research might benefit from interdisciplinary approaches, combining botanical, historical, and archaeological expertise to better understand ancient hygienic practices. Taking this into account, new investigations into the history of lavender can provide a fruitful foundation for advancements in modern hygienic medicine.

## Notes

### Competing interests

The authors declare that they have no competing interests.

### Authors’ ORCIDs

- Civilyte A: https://orcid.org/0000-0002-8793-7255
- Karanikola K: https://orcid.org/0009-0003-5743-8898
- Kramer A: https://orcid.org/0000-0003-4193-2149

### Funding

None. 
